# Experimental Evaluations on Seismic Performances of Porcelain and GFRP Composite UHV GIS Bushings

**DOI:** 10.3390/ma15114035

**Published:** 2022-06-06

**Authors:** Chang He, Ziwei He, Qiang Xie

**Affiliations:** 1 School of Civil Engineering, Central South University, Changsha 410075, China; heziwei@csu.edu.cn; 2 Jiangsu Key Laboratory of Engineering Mechanics, Southeast University, Nanjing 211189, China; 3 College of Civil Engineering, Tongji University, Shanghai 200092, China; qxie@tongji.edu.cn

**Keywords:** UHV GIS bushing, shaking table test, seismic performance, dynamic amplification effect, composite material

## Abstract

To evaluate the seismic performances of the ultra-high voltage (UHV) gas-insulated switchgear (GIS) bushings made by porcelain and glass fiber reinforced polymer (GFRP) composite materials, shaking table tests were conducted on the two full-scale GIS bushings. The dynamic characteristics and seismic responses of the two UHV GIS bushings were obtained. The experimental results indicated that the two UHV GIS bushings meet the seismic requirements in the corresponding standards. The supporting frame and bus canister amplify the seismic responses of the UHV GIS bushings. Under earthquakes, the GFRP composite UHV GIS bushing is safer than the porcelain bushing. In the seismic design of the electrical substation, the large seismic displacement of the GFRP composite UHV GIS bushings should be considered.

## 1. Introduction

There are many different pieces of electrical equipment in substations, e.g., bypass breakers, transformers, post-insulators, and disconnecting switches. The electrical equipment in the high voltage substation is usually made by porcelain ceramic or glass fiber reinforced polymer (GFRP) composite materials. The porcelain and GFRP composite materials are fragile materials. In earthquake events, the fragile materials are easily destroyed [1].

In past decades, many pieces of electrical equipment have been destroyed by earthquakes. In the Northridge earthquake in 1994, it was observed that the electrical equipment was cracked at their bottom cross sections [2,3]. In 2007, the Naketsu earthquake in the Niigata Prefecture destroyed a 500 kV transformer, causing a nuclear power plant to be deemed out of service [4]. The Wenchuan earthquake in 2008 destroyed more than 90 electrical substations [5]. Earthquake events in Chile [6], Haiti [7], and Mexico [1] in 2010 also destroyed many pieces of electrical equipment in substations and caused power interruptions. In the recent decade, the earthquakes also destroyed high voltage electrical equipment. Tohuku earthquake in 2011 destroyed 621 pieces of electrical equipment, including many bushings in 134 electrical substations [8]. In China, the Lushan earthquake [9], the Ludian earthquake, and the Jiiuzhaigou Valley earthquake occurred in 2013, 2014, and 2017, respectively, all destroying many pieces of electrical equipment, e.g., GIS bushings and transformer bushings. Moreover, in March 2022, the earthquake in Fukushima destroyed the power supply for 70,400 households. The disruption of the power system has made disaster relief and reconstruction inconvenient [10].

The seismic disaster investigations indicated that the porcelain bushing is one of the most vulnerable pieces of electrical equipment [11]. Many studies were conducted to investigate the seismic performance of the porcelain bushings. Shaking table tests were carried out on two 230-kV transformer bushings, and the retrofitting countermeasures were performed to prevent the oil-leakage under earthquakes [12,13]. In 1998 to 2013, the shaking table tests were carried out on the electrical bushings with different voltages, and it was found that the low voltage bushings can survive in the high-intensity earthquakes [14,15,16,17]. However, with the increase in the voltage, the electrical bushings were higher, heavier, and more slender. The seismic vulnerability of the electrical bushings with higher voltage increases [18].

With the development of material, the GFRP composite materials have been adopted in electrical equipment. The polymer composite bushing has a smaller weight compared with the porcelain counterparts. In 2016, experimental investigations were carried out by Moustafa and Mosalam to compare the mechanical performances of the porcelain and polymer composite post insulators. Considering the seismic performance, the polymer composite electrical equipment is more suitable for high-intensity areas [11]. Shaking table tests were conducted on 245-kV disconnecting switches. The weight of the porcelain disconnecting switch was 4.69 times that of the polymer composite switch, and the peak shear stress was 1.5~2.5 times of the polymer composite counterparts [19]. However, since the elastic modulus of the polymer material is much less than that of the porcelain, the deformation and seismic displacement of the polymer composite electrical equipment is usually greater [20]. The larger seismic displacement sometime makes the conductor connections inconvenient [21].

To improve the reliability of the electrical substation, the gas insulated switchgear (GIS) was used. Different pieces of electrical equipment, e.g., a disconnecting switch, a bypass breaker, and a lightning arrester, are contained in the GIS. To interconnect with other electrical equipment, GIS bushings, mounted on supporting structures, are needed in the substation. The supporting structure has great effects on the seismic performance of the electrical equipment [22,23].

In previous research, it was found that the GFRP composite electrical equipment is more suitable for a high-intensity seismic area. In the seismic performance evaluations on the electrical equipment, investigations were carried out on porcelain GIS bushings. With the increase in the voltage, the UHV GIS bushings may be more vulnerable. In the recent decade, the GIS bushings made by polymer composite materials have been used in electrical substations. However, there are few studies on the seismic performance of the GFRP composite GIS bushings. To evaluate the seismic performances of the GIS bushings made by porcelain and polymer composite materials, this paper concentrates on the shaking table tests on two full-scale UHV GIS bushings made by the two different materials. The supporting structure of the two GIS bushings were the same. The dynamic characteristics, accelerations, and strain responses of the two bushings were compared, and the seismic performances of the two bushings were evaluated. Further, by comparing the seismic responses of the two UHV GIS bushings mounted on the same supporting structure, the effects of the materials on the seismic performance of the post electrical equipment were investigated.

## 2. Description of Specimens

### 2.1. UHV GIS Bushings

**Figure 1:**
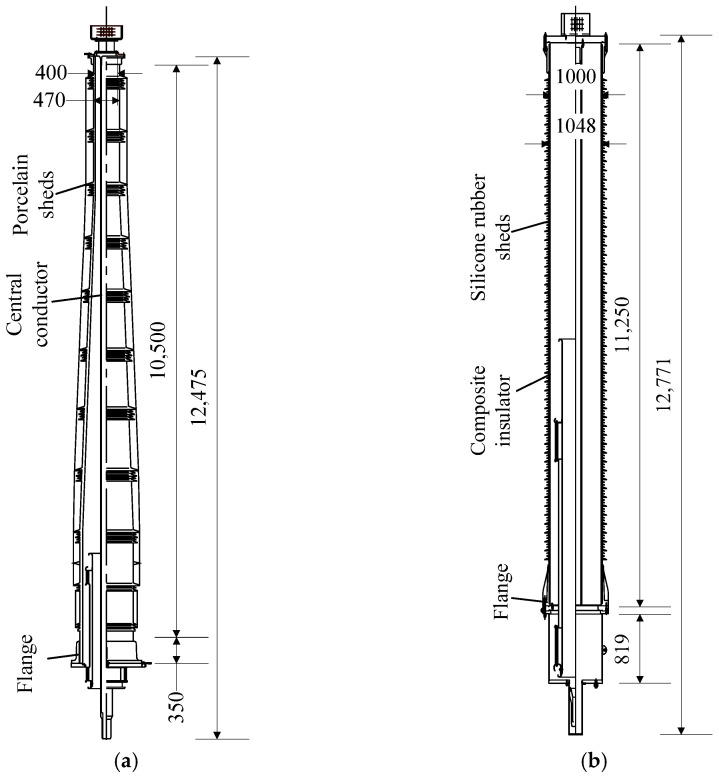
Configurations and dimensions of the two UHV GIS bushings (Unit: mm). (**a**) Porcelain bushing; (**b**) GFRP composite bushing.

The porcelain bushing is shown in Figure 1a. The porcelain bushing was mounted in a copper flange, and the porcelain bushing and flange were connected by cement. To transmit the electrical current, an aluminum central conductor was installed in the bushing. A terminal pad was installed at the top of the bushing to connect the conductor. Besides, a grading ring was mounted on the bushing to maintain the electromagnetic field around the terminal pad, and SF_6_ insulating gas were filled in the bushing for internal insulation, with an air pressure of 0.45 MPa. The total length of the porcelain bushing is 12.475 m, with a length of the porcelain part of 10.5 m. The total weight of the porcelain bushing is 6300 kg, and the location of the centre of gravity is distanced to the flange with 4.45 m. At the bottom cross section of the porcelain bushing, the outer and inner diameters of the porcelain bushing are 860 mm and 960 mm, respectively, and the corresponding diameters of the cross section at the top of the porcelain bushing are 400 mm and 470 mm, respectively. According to the manufacturer, the ultimate bending strength and elastic modulus of the porcelain are 28 MPa and 88 GPa, respectively.

The polymer composite bushing is shown in Figure 1b. The total length of the composite bushing is 12.27 m, and the length of the composite polymer composite insulator is 11.25 m. Similar to the porcelain counterpart, the polymer composite insulator is installed in an aluminum flange, and a central conductor is installed in the insulator, with a 0.5-MPa air pressure SF_6_. To increase the outer insulating distance, the sheds made by silicone rubber are set around the outer surface of the GFRP composite insulator. The inner and outer diameters of the composite bushing are 1000 mm and 1048 mm, respectively. Moreover, the total weight of the composite bushing is 4400 kg, and the centre of gravity is a 5.6 m distance to the flange. Although the diameter of the composite bushing is greater than the porcelain bushing, the former one is still lighter. Besides, according to the manufacturer, the ultimate bending strength of the composite material is 75 MPa, which is much greater than that of the porcelain. The elastic modulus of the composite material is 17 GPa, which is much lower than 88 GPa, the corresponding value of the porcelain.

### 2.2. Supporting Frame and Bus Canister

**Figure 2:**
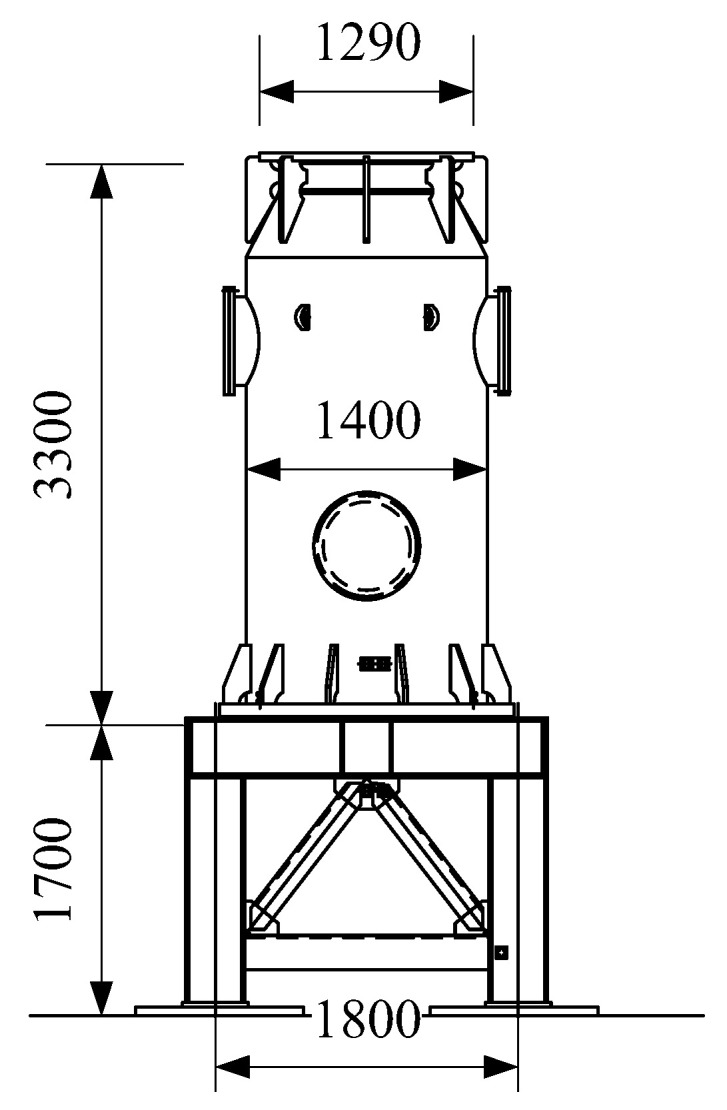
Configuration and dimensions of the supporting frame and bus canister (Unit: mm).

In an electrical substation, the GIS bushing is mounted on a bus canister. The central conductor of the bushing is connected with the electrical equipment in the bus canister. Besides, the bus canister is mounted on a steel supporting frame. The configurations of the supporting frame and bus canister are shown in Figure 2.

The height of the steel supporting frame is 1.7 m, with a width of 1.8 m. The columns and beams of the supporting frame are H350 × 350 × 12 × 19 wide flange H-shaped steel. The height and width of the steel columns and beams are 350 mm, and the thicknesses of the webs and flanges are 12 mm and 19 mm, respectively. The cross section of the inclined braces is L140 × 14, and the cross section of the horizontal braces is L200 × 14. Besides, the weight of the steel supporting braces is 4102 kg.

To connect the electrical equipment in the GIS, the bus canister is needed. In this type of the UHV GIS bushings, the height of the steel bus canister is 3.3 m, with an outer diameter of 1.4 m, and the thickness of the steel tube is 16 mm. The total weight of the bus canister is 2450 kg. In the UHV GIS bushings, the bushing, bus canister, and supporting frames are connected by flanges (Figure 2).

## 3. Test Procedures and Instrumentations

### 3.1. Seismic Requirements and Earthquake Ground Motion

**Figure 3:**
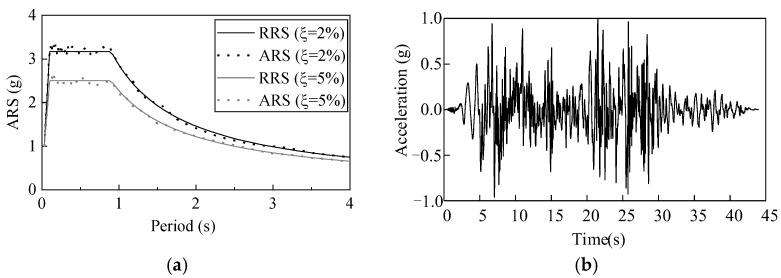
Response spectra and acceleration time history of synthetic earthquake ground motion. (**a**) Comparison of the ARS and RRS with different damping ratios (ξ); (**b**) Acceleration time history of the synthetic earthquake ground motion.

A seismic required response spectra (RRS) was stipulated in the Chinese Q/GDW11132-2013 standard to qualify the seismic performance of the UHV electrical equipment (Figure 3a) [24]. In the shaking table tests, the acceleration response spectra (ARS) of the earthquake ground motion should accommodate the RRS. Besides, the Q/GDW11132-2013 standard stipulates that the peak ground acceleration (PGA) in the qualification should be determined by the site-specific seismic hazard evaluation with a 2% probability of exceedance in 50 years [25]. In the Q/GDW11132-2013 standard, four types of site profile were considered with different predominant periods. To evaluate the seismic performances of the porcelain and GFRP composite UHV GIS bushings, the predominant period of the ARS was set as 0.9 s, the maximum predominant period in the standard [24]. Thus, the qualification results of the tests can be adopted for any type of site profile.

In the shaking table tests, a synthetic earthquake ground motion was used (Figure 3b). The synthetic earthquake ground motion was modulated from the record of Landers earthquake in 1992. With damping ratios of 2% and 5%, the ARS of the synthetic time history accommodates the RRS well (Figure 3a). This synthetic time history has been used to evaluate the seismic performances of different UHV electrical equipment [18,20,23]. Moreover, since the UHV GIS bushings are installed vertically in sites, according to the stipulation in the Q/GDW11132-2013 standard, the UHV GIS bushings can be tested uniaxially [24].

### 3.2. Test Procedures

The test scenarios of the porcelain and GFRP composite UHV GIS bushings are listed in Table 1. To detect the dynamic characteristics of the two UHV GIS bushings, a white noise acceleration time history was used in the shaking table tests. Besides, after each seismic test, the white noise acceleration time history was also adopted to detect whether there was any structural damage in the UHV GIS bushings.

The mass and stiffness of the specimens affect the dynamic properties of the shaking table. Thus, before the seismic tests, iterations on the shaking table control systems were needed to minimize the tolerances between the input synthetic time history and the output acceleration records at the shaking table. To iterate the shaking table control systems, the tests under the synthetic time history with a low intensity (PGA = 0.15 g) were conducted three times.

To evaluate the seismic performance of the porcelain UHV GIS bushing, the PGA of the synthetic earthquake motion was 0.4 g, the maximum PGA stipulated in the Q/GDW11132-2013 standard. The UHV electrical substations are the critical nodes in the power grid. For the important projects, the standard stipulates that seismic safety evaluations are needed. If the maximum PGA recommended by the seismic safety evaluation report was greater than the maximum value in the standard, the maximum PGA in the seismic tests should be updated to the PGA recommended in the seismic safety evaluation report. For the GFRP composite bushing, the maximum PGA was set as 0.5 g.

**Figure 4:**
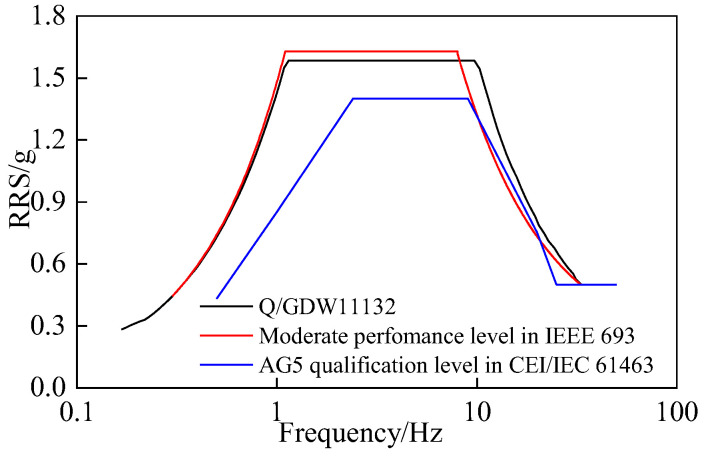
Comparison of the RRS in the three standards after considering the corresponding amplification factor (damping ratio = 2%).

Besides, the RRS in the IEEE 693 standard [26] and the IEC 61463 standard [27] are compared in Figure 4. For the moderate level in the IEEE 693 standard and the AG5 level in the IEC 61463 standard, the PGA was 0.5 g. In Figure 4, the RRS in the IEEE 693 and Q/GDW11132-2013 standard were similar, and are higher than those of the IEC 61463 standard.

### 3.3. Test Instrumentations

**Figure 5:**
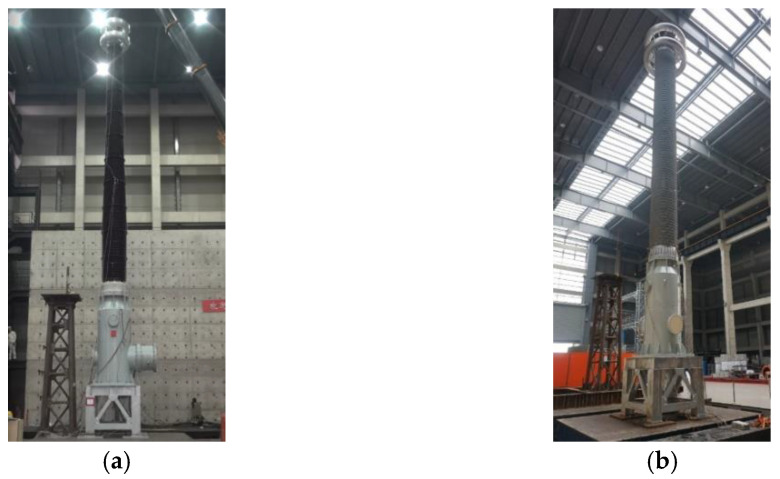
UHV GIS bushings mounted on shaking table. (**a**) Porcelain bushing; (**b**) GFRP composite bushing.

**Figure 6:**
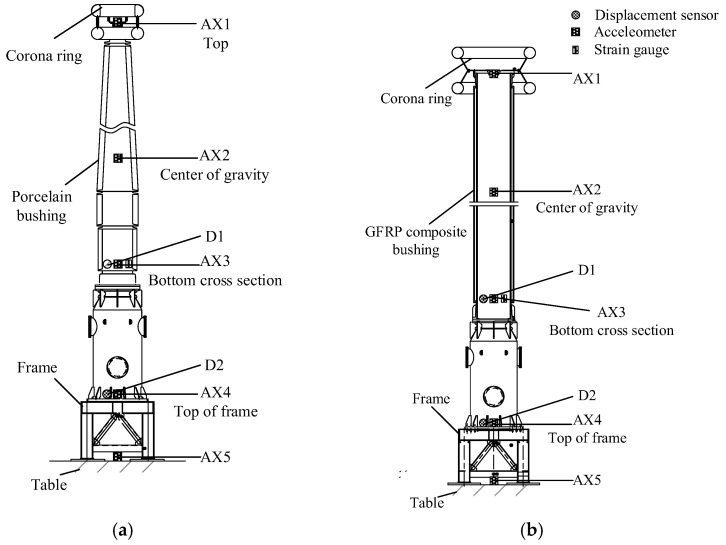
Test instrumentations of the two UHV GIS bushings. (**a**) Porcelain bushing; (**b**) GFRP composite bushing.

The UHV GIS bushings mounted on the shaking table are shown in Figure 5. The arrangement of the sensors on the two UHV GIS bushings are shown in Figure 6. To obtain the dynamic responses and characteristics of the UHV GIS bushings, the accelerometers were arranged at the top and the centers of gravity of the two bushings. The acceleration amplification factor (AAF) is defined as the ratio of the maximum acceleration at the flange of the bushing to the PGA. The Q/GDW11132-2013 standard recommends that the AAF should not be greater than 1.4 for the UHV electrical equipment. To investigate the amplification effects of the supporting frame and bus canister, two accelerometers were arranged at the top of the supporting frame and bus canister, respectively.

**Figure 7:**
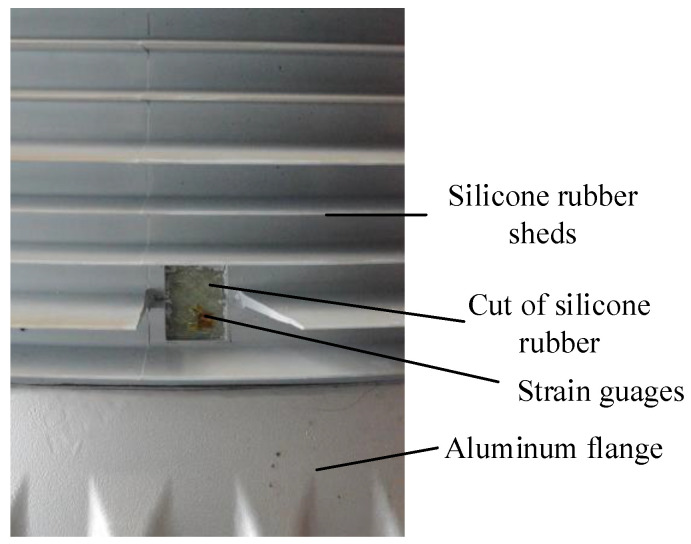
Cut of the silicone rubber in the GFRP composite bushing.

In past earthquakes, the electrical equipment is usually cracked at its bottom cross section [12,13,14,15,20,28]. Therefore, strain gauges were arranged at the bottom cross sections of the porcelain and GFRP composite UHV GIS bushings, along the longitudinal axis of the bushing. To measure the strain responses of the GFRP composite bushing, the silicone rubber near the instrumentations were cut (Figure 7) and the strain gauges were installed at the GFRP material.

The seismic displacement at the top of the electrical equipment affects the interactions between the equipment and conductor, and the impact effects in the conductor may destroy the interconnected electrical equipment [28]. Since the heights of the two specimens were higher than 17 m, it is difficult to construct a frame to install the displacement sensors. In the shaking table tests, the displacement responses of the UHV GIS bushings were obtained by double integrations on the acceleration records. To validate the integration method, two displacement sensors were installed at the top of the supporting frame and the bottom of the bushings (the top of the bus canister).

## 4. Experimental Result Analyses

### 4.1. Dynamic Characteristics

The dynamic characteristics are critical parameters for the seismic evaluations. Besides, the comparisons between the dynamic characteristics before and after the seismic evaluation tests can reflect the structural damage in the two UHV GIS bushings.

**Figure 8:**
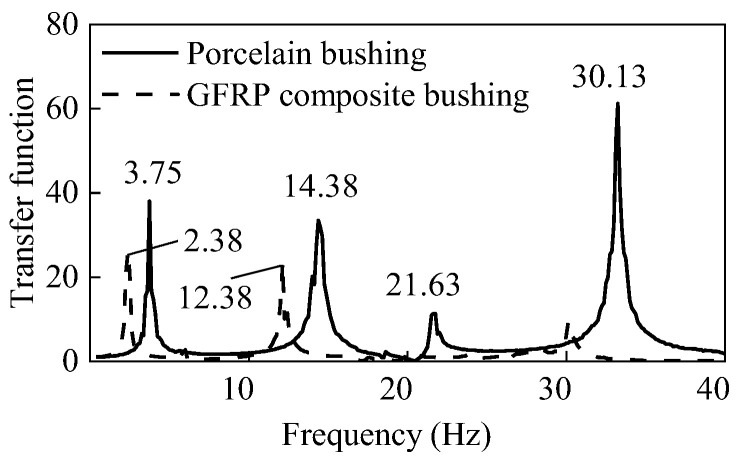
Transfer functions of the two UHV GIS bushings under the first white noise tests.

Under the first white noise tests, the transfer functions of the two UHV GIS bushings are shown in Figure 8. The first two-order frequencies of the GFRP composite bushing were 2.38 Hz and 12.38 Hz, respectively. Moreover, the first two-order frequencies of the porcelain counterpart were 3.75 Hz and 14.38 Hz. Since the elastic modulus of the porcelain was much greater than that of the GFRP composite material, the frequencies of the porcelain UHV GIS bushings were higher.

**Figure 9:**
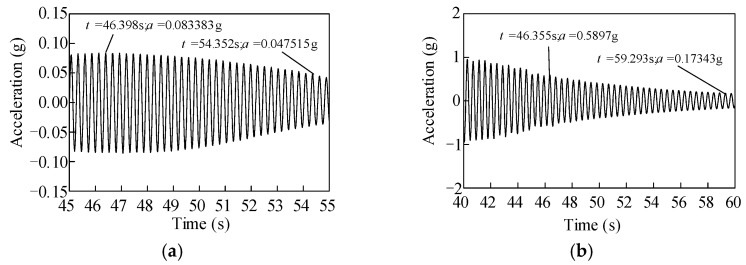
Acceleration decrement of the two bushings after the white noise test stopped. (**a**) Porcelain UHV GIS bushing; (**b**) GFRP composite UHV GIS bushing.

The logarithmic decrement method was adopted to evaluate the damping ratios of the two full-size UHV GIS bushings. When the excitations of the first white noises stopped, the acceleration time histories at the top of the two UHV GIS bushings are shown in Figure 9. For the porcelain bushing, the maximum acceleration responses decreased from 0.0834 g to 0.0475 g in 33 vibration cycles, and the damping ratio of the porcelain UHV GIS bushing was 0.3%. Similarly, after a 37-cycle decrement, the maximum acceleration at the top of the GFRP composite UHV GIS bushing decreased from 0.5897 g to 0.1734 g, and the damping ratio was 0.5% according to the logarithmic decrement method. The damping ratios of both of the two UHV GIS bushings were smaller than the value of 2%, recommended by the Q/GDW11132-2013 standard. To evaluate the seismic performances of the UHV GIS bushings by theoretical or numerical methods, the smaller damping ratios should be obtained to get safer evaluation results.

### 4.2. Seismic Performance Evaluations

#### 4.2.1. Strength Evaluation

To evaluate the seismic performances of the two UHV GIS bushings, according to the Q/GDW11132-2013 standard, the maximum stresses of the bushing and the resonance frequency change after the seismic test have to be considered [24].

**Figure 10:**
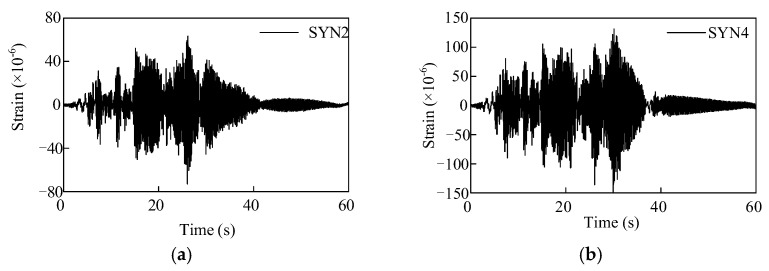
Seismic strain responses of the porcelain UHV GIS bushing. (**a**) PGA 0.15 g; (**b**) PGA 0.4 g.

**Figure 11:**
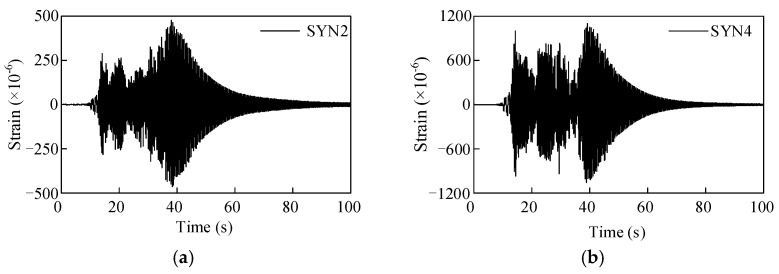
Seismic strain responses of the GFRP composite UHV GIS bushing. (**a**) PGA 0.15 g; (**b**) PGA 0.5 g.

**Figure 12:**
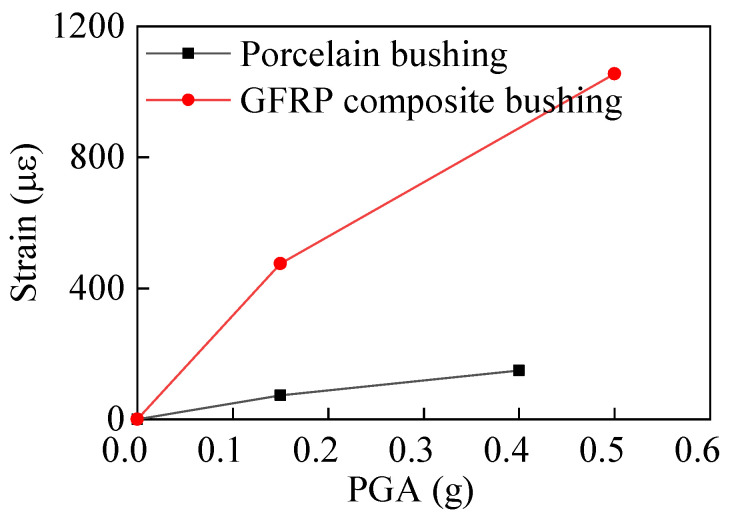
Seismic strain responses of the porcelain and GFRP composite UHV GIS bushings.

The strain responses of the porcelain GIS bushings under different intensity earthquakes are shown in Figure 10. Moreover, the strain responses of the GFRP composite bushing with different earthquake intensities are shown in Figure 11. The maximum strain responses of the two bushings with respect to the PGAs are shown in Figure 12. According to Figure 12, when the PGA was less than 0.4 g, the strain responses were almost in a linear relationship, and the seismic responses of the porcelain UHV GIS bushing were in a linear range. However, for the GFRP composite bushing, when the PGA was less than 0.5 g, the nonlinear behaviour can be observed. The strain responses under 0.5-g earthquake were lower than the linear prediction value. Under the high-intensity earthquake (PGA = 0.5 g), the damage occurred in the material of the composite UHV GIS bushing. It was the reason that the nonlinear phenomenon occurred. In the theoretical or numerical analyses on the GFRP composite UHV GIS bushing, the damage constitutive model of the composite material should be adopted. However, to the authors’ best knowledge, there is not a damage-considered constitutive model for the GFRP composite material. Further investigations are needed to investigate the nonlinear mechanisms of the GFRP composite bushing.

Under the synthetic earthquake ground motion, with a PGA of 0.4 g, the maximum tensile strain responses of the porcelain UHV GIS bushing was 139.84 με, considering the elastic modulus of the porcelain provided by the manufacturer, the maximum tensile stress of the porcelain was 12.31 MPa, which was less than the ultimate strength, 28 MPa, of the porcelain. Under the synthetic earthquake record with a PGA of 0.5 g, the maximum tensile strain of the GFRP composite UHV GIS bushing was 1079.25 με. Since the elastic modulus of the GFRP material was 17 GPa, the maximum tensile stress was 18.35 MPa. In the shaking table tests, the maximum stresses of the two UHV GIS bushings were all less than their ultimate strengths, and the bushings would not damage in the laboratory. This conclusion was consistent with the phenomenon in the laboratory.

The Q/GDW11132-2013 standard stipulates that the stresses generated by the wind loads and inner air pressures should be considered in the seismic evaluation. And the load combination is:

(1) $$\sigma =\sigma_{E}+0.25\sigma_{W}+\sigma_{p}$$

In Equation (1), *σ* denotes the maximum stress of the electrical equipment after load combination. *σ_E_*, *σ_W_*, and *σ_p_* are the maximum tensile strength generated by the earthquake, wind load, and inner air pressure, respectively. After the load combinations, the maximum stresses of the porcelain and polymer UHV GIS bushings were 14.71 MPa and 25.13 MPa, respectively.

The stress safety factor is defined as the ultimate strength of the material to the maximum stress response of the electrical equipment. Therefore, the safety factors of the porcelain and GFRP composite UHV GIS bushings were 1.90 and 2.98. In the GB 50260 and Q/GDW11132-2013 standards [24,29], the safety factor of the porcelain electrical equipment should be greater than 1.67. Besides, in the IEEE 693 standard it was recommended that the safety factor of the GFRP composite electrical equipment should be greater than 2.0 [26]. In the shaking table tests, the safety factors of the two bushings met the requirements in the corresponding standards.

#### 4.2.2. Dynamic Characteristics Change

**Figure 13:**
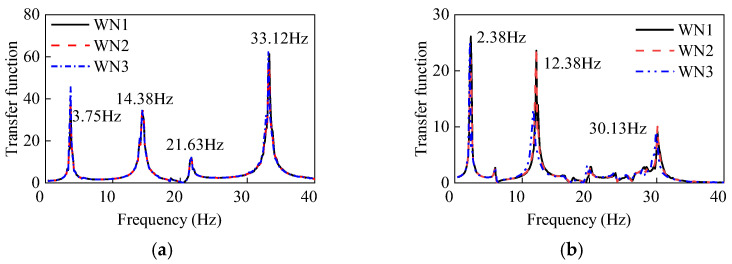
Transfer functions of the two UHV GIS bushings under the three white noise tests. (**a**) Porcelain UHV GIS bushing; (**b**) GFRP composite UHV GIS bushing.

According to Table 1, after each seismic test under the synthetic time history, the white noise tests were carried out to detect whether there were any structural damages in the UHV GIS bushings. Under the three white noise tests (WN1, WN2, and WN3), the transfer functions of the three tests for the two UHV GIS bushings are shown in Figure 13, and the first two-order frequencies obtained by each white noise test are listed in Table 2. In Figure 13a, the three transfer functions of the porcelain bushings were the same, and the first two-order frequencies of the porcelain UHV GIS bushing had not been changed. These results indicated that there was no structural damage in the porcelain UHV GIS bushing. For the GFRP composite bushing, the three transfer functions of the white noises were similar. According to Figure 13b and Table 2, after the Test SYN1~3, the transfer function curves and the first two-order of the GFRP composite UHV GIS bushing did not change. However, after the high-intensity earthquake (SYN4), the first two-order frequencies of the GFRP composite UHV GIS bushing decreased by 1.26% and 5.57%, respectively. The frequencies of the GFRP composite electrical equipment usually decreased after seismic tests [28]. In engineering practice, if the frequencies decreasing of the GFRP composite electrical equipment was less than 10%, the GFRP composite electrical bushing met the frequency decreasing requirements. Additionally, the phenomenon that the frequencies of the GFRP composite bushing decreased was consistent with the nonlinear behavior of the strain responses. The structural damage changed the resonance frequencies and generated the nonlinear responses.

In summary, the safety factors and the dynamic characteristics of the porcelain and GFRP composite UHV GIS bushings met the requirements in the corresponding standards. The seismic performances of the two UHV GIS bushings were qualified.

### 4.3. Acceleration and Displacement Responses

**Figure 14:**
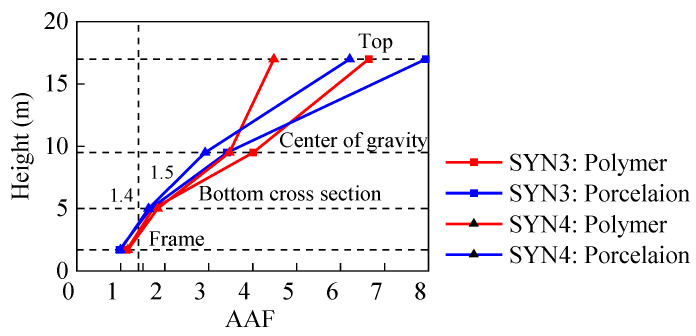
AAFs of the porcelain and GFRP composite UHV GIS bushings.

The acceleration amplification factor (AAF) is defined as the ratio of the maximum acceleration at the electrical equipment to the PGA. The AAFs of the porcelain and GFRP composite UHV GIS bushings are shown in Figure 14. Comparing the curves of the UHV GIS bushings with different seismic intensities, the AAFs under the low-intensity earthquakes were greater than those under the high-intensity, and the decreasing ratios are listed in Table 3. With a PGA of 0.15 g, the vibration amplitude of the UHV GIS bushings were lower. Thus, the damping ratios of the UHV GIS bushings under the low intensity were smaller, which generated the greater AAFs.

At the centers of gravity, since the elastic modulus of the GFRP composite material was lower than that of the porcelain, the AAFs of the GFRP composite bushings were greater than those of the porcelain counterpart. The diameter of the porcelain UHV GIS bushings decreased at the top part. Thus, the AAFs increased at the top of the porcelain UHV GIS bushing.

The AAFs of the supporting frame reflects the dynamic interactions between the electrical equipment and its supporting structure. Since the dynamic effects, the supporting structure amplifies the seismic responses of the electrical equipment mounted on the supporting structure. This phenomenon was observed in previous research [18]. The GB 50260 standard and the Q/GDW11132-2013 standards recommended that the amplification factor of the supporting structure should not be greater than 1.2 and 1.4, respectively [24,29]. According to Figure 14, the AAFs of the steel frame and bus canister of the porcelain bushing were smaller than those of the GFRP composite bushing. Moreover, the AAFs at the top of the bus canister were all greater than 1.4, the maximum AAF recommended by the standards. Thus, the lateral stiffness of the bus canister should be increased to decrease the AAF and the seismic responses of the UHV GIS bushings mounted on it.

The seismic displacements of the electrical equipment affect the slackness of the conductor interconnected with the electrical equipment. Under different intensities of earthquakes, the seismic displacements at the top of the porcelain and GFRP composite UHV GIS bushings are listed in Table 4. In Table 4, the seismic displacements of the porcelain UHV GIS bushings were much smaller than those of the GFRP composite bushings, even though the diameter of the GFRP bushings was greater and the mass of it was lighter than that of the porcelain UHV GIS bushing.

## 5. Conclusions

In this paper, shaking table tests were carried out on two full-scale UHV GIS bushings to evaluate their seismic performances. The two GIS bushings were made by porcelain and GFRP composite materials, respectively. The dynamic characteristics, seismic responses, and dynamic property changes of the UHV GIS bushings after the seismic tests were analyzed, and the effects of the two materials on the dynamic characteristics and seismic responses of the UHV GIS bushings were compared. The following conclusions can be drawn:

- The UHV GIS bushings are slender structures. The resonance frequencies of the GFRP composite bushing are lower than those of the porcelain counterpart.
- The damping ratios of the UHV GIS bushings are much less than the recommended value in the corresponding standards. To evaluate the seismic performances of the UHV GIS bushings and in the seismic design of the electrical substation, the lower damping ratios should be adopted.
- Considering the wind loads and inner air pressures, for the porcelain bushing under the earthquake with a PGA of 0.4 g and the GFRP composite bushing with a PGA of 0.5 g, the two UHV GIS bushings all meet the seismic requirements in the corresponding standards. The two UHV GIS bushings can be adopted in the UHV projects in the high-intensity seismic areas.
- The safety factor of the GFRP composite bushing is greater than that of the porcelain counterpart. However, since the elastic modulus of the GFRP composite material is lower, the seismic displacement of the GFRP composite bushing is much greater than that of the porcelain bushing. In the seismic design of the substation, the length of the interconnected conductor should satisfy the seismic displacement of the UHV GIS bushing.

## Figures and Tables

**Table 1:** Test scenarios of the porcelain and GFRP composite bushings.

Test Serial Number	Earthquake Ground Motion	PGA/g	Purpose
WN1	White noise.	0.075	Detecting the dynamic characteristics of the UHV GIS bushings.
SYN1~3	Synthetic time history.	0.15	Iterating the control system of the shaking table to minimize the tolerances between the input and output acceleration.
WN2	White noise.	0.075	Detecting whether there was any structural damage in the UHV GIS bushings.
SYN4	Synthetic time history.	0.4/0.5 ^1^	Seismic performances evaluations.
WN3	White noise.	0.075	Detecting whether there was any structural damage in the UHV GIS bushings.

^1^ The maximum PGA for the porcelain and GFRP composite UHV GIS bushings were 0.4 g and 0.5 g, respectively.

**Table 2:** First two-order frequencies of the two bushings under the three white noise tests (Unit: Hz).

Test	Porcelain Bushing		GFRP Composite Bushing	
	1st Frequency	2nd Frequency	1st Frequency	2nd Frequency
WN1	3.75	14.38	2.38	12.38
WN2	3.75	14.38	2.38	12.38
WN3	3.75	14.38	2.25	11.69

**Table 3:** AAFs and the decreasing ratios of the porcelain and GFRP composite UHV GIS bushings.

Locations	Porcelain Bushing			GFRP Composite Bushing		
	SYN3	SYN4	Decreasing Ratio (%)	SYN3	SYN4	Decreasing Ratio (%)
Top of frame	1.00	1.00	0	1.14	1.16	1.75
Top of canister	1.68	1.63	−1.79	1.74	1.84	5.75
Center of gravity	3.42	2.92	−14.62	4.01	3.47	−13.47
Top of bushing	7.94	6.21	−21.79	6.64	4.48	−32.53

**Table 4:** Seismic displacements of the two bushings (Unit: mm).

Porcelain Bushing		GFRP Composite Bushing	
SYN3	SYN4	SYN3	SYN4
16.89	44.85	40.02	122.29

## Data Availability

The data provided in this study could be released upon reasonable request.
